# Urinary Dopamine as a Potential Index of the Transport Activity of Multidrug and Toxin Extrusion in the Kidney

**DOI:** 10.3390/ijms17081228

**Published:** 2016-07-30

**Authors:** Moto Kajiwara, Tsuyoshi Ban, Kazuo Matsubara, Yoichi Nakanishi, Satohiro Masuda

**Affiliations:** 1 Department of Pharmacy, Kyushu University Hospital, 3-1-1 Maidashi, Higashi-ku, Fukuoka 812-8582, Japan; satomsdb@pharm.med.kyushu-u.ac.jp; 2 Department of Clinical Pharmacology and Therapeutics, Kyoto University Hospital, 54 Kawaharacho, Shogoin, Sakyo-ku, Kyoto 606-8507, Japan; ban.tsuyoshi.73v@gmail.com (T.B.); kmatsuba@kuhp.kyoto-u.ac.jp (K.M.); 3 Research Institute for Diseases of the Chest, Graduate School of Medical Sciences, Kyushu University, 3-1-1 Maidashi, Higashi-ku, Fukuoka 812-8582, Japan; yoichi@kokyu.med.kyushu-u.ac.jp

**Keywords:** dopamine, MATE, natriuresis, imatinib, fluid retention

## Abstract

Dopamine is a cationic natriuretic catecholamine synthesized in proximal tubular cells (PTCs) of the kidney before secretion into the lumen, a key site of its action. However, the molecular mechanisms underlying dopamine secretion into the lumen remain unclear. Multidrug and toxin extrusion (MATE) is a H^+^/organic cation antiporter that is highly expressed in the brush border membrane of PTCs and mediates the efflux of organic cations, including metformin and cisplatin, from the epithelial cells into the urine. Therefore, we hypothesized that MATE mediates dopamine secretion, a cationic catecholamine, into the tubule lumen, thereby regulating natriuresis. Here, we show that [^3^H]dopamine uptake in human (h) MATE1-, hMATE-2K- and mouse (m) MATE-expressing cells exhibited saturable kinetics. Fluid retention and decreased urinary excretion of dopamine and Na^+^ were observed in *Mate1*-knockout mice compared to that in wild-type mice. Imatinib, a MATE inhibitor, inhibited [^3^H]dopamine uptake by hMATE1-, hMATE2-K- and mMATE1-expressing cells in a concentration-dependent manner. At clinically-relevant concentrations, imatinib inhibited [^3^H]dopamine uptake by hMATE1- and hMATE2-K-expressing cells. The urinary excretion of dopamine and Na^+^ decreased and fluid retention occurred in imatinib-treated mice. In conclusion, MATE transporters secrete renally-synthesized dopamine, and therefore, urinary dopamine has the potential to be an index of the MATE transporter activity.

## 1. Introduction

Dopamine is a cationic natriuretic catecholamine. Excretion of this hormone and urinary Na^+^ concentration are both increased by Na^+^ intake and acute saline infusion [1,2,3]. Dopamine receptors are classified into two groups, D1-like (D1 and D5) and D2-like (D2, D3 and D4), which are both expressed in the kidney [4]. After moderate Na^+^ loading, more than 50% of the incremental urinary Na^+^ excretion is attributable to the stimulation of D1-like receptors with renally-synthesized dopamine [1,5]. The subsequent increase in urinary Na^+^ excretion is accompanied by an increase in urine output [1,5]. A study in humans indicates that urinary dopamine is derived from the kidney since plasma dopamine concentration (0.43 ± 0.06 nM) and total plasma volume are insufficient to achieve the almost 1000-fold greater urinary dopamine concentration (0.63 ± 0.17 µM) [6]. Renal dopamine synthesis is restricted to proximal tubular cells (PTCs), which internalize the circulating and glomerular-filtered forms of l-dihydroxyphenylalanine (l-DOPA) via dimers of the 4F2 heavy chain/l-type amino acid transporter 2 and related to B°^,+^ amino acid transporter/B°^,+^-type amino acid transporter, respectively [7,8]. PTCs express aromatic amino acid decarboxylase (AADC) [9], which converts internalized l-DOPA to dopamine. Renally-synthesized dopamine is secreted into the tubular lumen and acts at dopamine receptors expressed in multiple nephron segments, thereby inhibiting the Na^+^ transport activity of various targets, including the Na^+^/H^+^ exchanger (NHE)-1, NHE3, Na^+^/P cotransporter IIa, Na^+^/HCO_3_^−^ cotransporter, Cl^−^/HCO_3_^−^ exchanger and Na^+^/K^+^ ATPase [10]. Although dopamine secretion from PTCs to the lumen is a key step in natriuresis, the molecular mechanisms underlying dopamine secretion remain unknown.

In the current study, we focused on multidrug and toxin extrusion (MATE), which is also known as SLC47A, a candidate transporter that provides dopamine into the proximal tubular lumen (PTL). MATE is a H^+^/organic cation antiporter that is highly expressed in brush border membranes of PTCs and mediates the tubular secretion of organic cations by using a H^+^ gradient [11,12,13]. MATE1 and MATE2-K are expressed in the human kidney tissue, whereas Mate1 is expressed in mice [13,14]. Organic cations, such as tetraethylammonium, cimetidine, metformin, creatinine and varenicline, are typical substrates for MATE transporters [15,16]. Because they transport several clinically-important drugs, MATE1 and MATE2-K are included in the battery of the in vitro tests used in the process of new drug development, as recommended by the International Transporter Consortium, the European Medicines Agency and the U.S. Food and Drug Administration [17,18,19]. MATE transporters play a critical role in the excretion of metformin, a biguanide antidiabetic drug that is mainly excreted in the urine in a non-metabolized form. Tubular secretion plays a major role in this process since the renal metformin clearance is almost five-fold greater than its creatinine clearance is [20]. In *Mate1*-knockout mice, the area under the blood concentration-time curve of metformin at 60 min and renal secretory clearance of metformin were two-fold higher and 86% lower, respectively, than the respective values in wild-type mice were [14]. An in vitro uptake study showed that tyrosine kinase inhibitors blocked [^14^C]metformin uptake by human MATE transporters; imatinib was the most effective agent, which displayed the lowest half-maximal inhibitory concentration (IC_50_) of all of the drugs tested [21].

In this study, we carried out uptake experiments and acute saline infusion experiments in *Mate1*-knockout mice and MATE inhibitor (imatinib)-treated mice. The results indicate that MATE facilitated the transfer of dopamine into the PTL and promoted natriuresis, and therefore, urinary dopamine has potential usefulness as a noninvasive index of transport activity of MATE in the kidney.

## 2. Results

### 2.1. Dopamine Transport Is Mediated by Multidrug and Toxin Extrusion (MATE)

**Figure 1:**
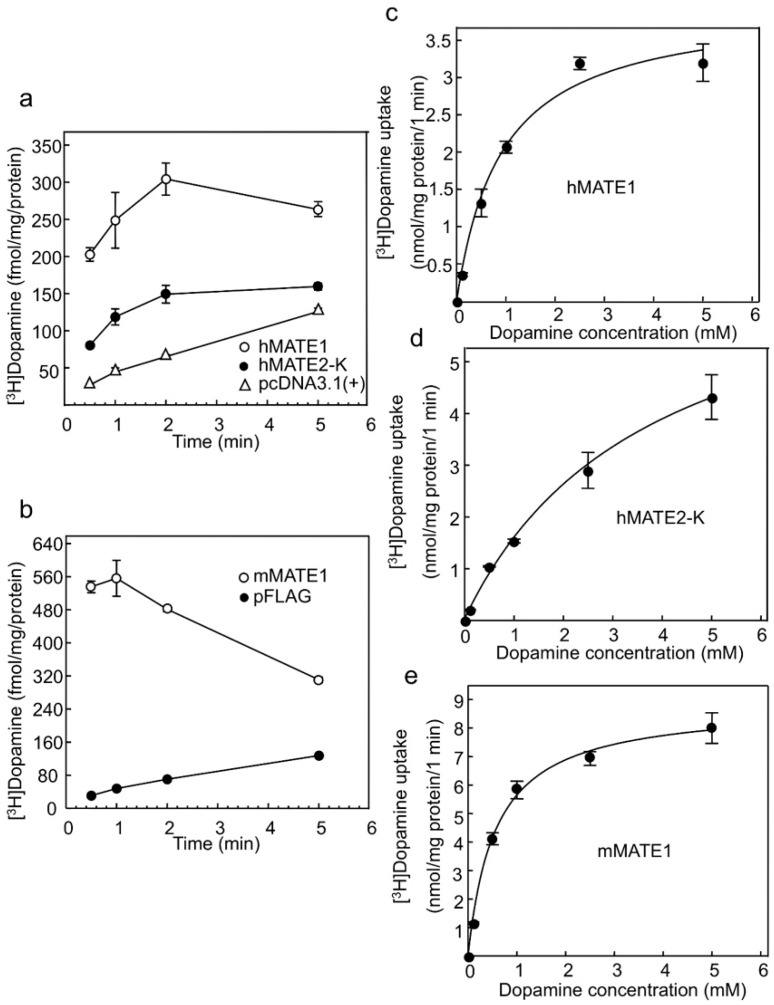
Characteristics of dopamine transport mediated by human multidrug and toxin extrusion (hMATE)1-, hMATE2-K- and mouse (m) MATE1-expressing cells (**a**) Time course of [^3^H]dopamine uptake by hMATE1- (Ο) and hMATE2-K- (●) expressing cells. pcDNA3.1(+) (Δ) represents cells transfected with empty vector. Each set of points represents uptake values at 0.5, 1, 2 and 5 min. The level of [^3^H]dopamine uptake at all time points in the presence of MATE transporters was significantly higher than that of the controls was, *p* < 0.01 (*n* = 3); (**b**) Time course of [^3^H]dopamine uptake by mMATE1- (Ο) expressing cells. pFLAG (●) is the empty vector. Each set of points represents uptake values at 0.5, 1, 2 and 5 min. [^3^H]dopamine uptake in the cells expressing MATE transporters was significantly higher than that of controls at all time points, *p* < 0.01 (*n* = 3); (**c**) Concentration-dependent uptake of [^3^H]dopamine by hMATE1-expressing cells (*n* = 3); (**d**) Concentration-dependent uptake of [^3^H]dopamine by hMATE2-K-expressing cells (*n* = 3); (**e**) Concentration-dependent uptake of [^3^H]dopamine by mMATE1-expressing cells (*n* = 3). For analyses in (**c**–**e**), dopamine concentrations were 8.2 × 10^−5^, 0.1, 0.5, 1, 2.5 and 5 mM.

To examine whether dopamine is a substrate of MATE transporters, we carried out uptake experiments, which are frequently used to evaluate MATE transport properties [21,22,23,24,25]. Significant uptake of [^3^H]dopamine by human (h) MATE1 (Figure 1a), hMATE2-K (Figure 1a) and mouse (m) MATE1 (Figure 1b) compared to that of the cells transfected with an empty vector was observed at each time point (*p* < 0.01). The transport characteristics of MATE transporters were examined at 1 min in subsequent experiments because of technical limitations and reproducibility. An “overshoot” was observed because the driving force (an outward H^+^ gradient for MATE) was depleted during the uptake experiments, and substrate backflow occurred [26,27]. To estimate the kinetic parameters for [^3^H]dopamine uptake by hMATE1, hMATE2-K and mMATE1, concentration-dependent uptake was examined, and the dopamine uptake by all three transporters exhibited saturable kinetics, following the Michaelis–Menten equation (Figure 1c,d,e). The apparent maximal uptake velocity (*V*_max_), Michaelis–Menten constant (*K*m) and *V*_max_/*K*m values are summarized in Table 1. The rank order of the [^3^H]dopamine transport activity (*V*_max_/*K*m) was mMATE1 > hMATE1, hMATE2-K.

### 2.2. Effects of Mate1 Knockout on Urinary Dopamine and Na^+^ Excretion in Mice

**Figure 2:**
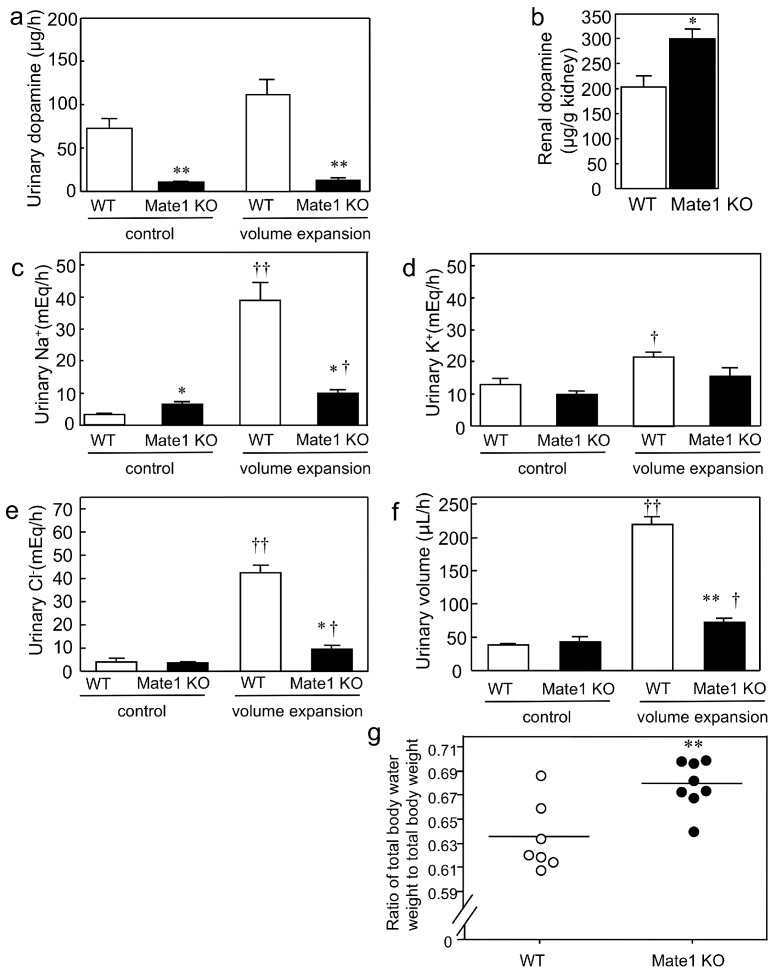
Effect of multidrug and toxin extrusion (*Mate1*) knockout on natriuresis resulting from renally-synthesized dopamine and fluid retention in mice. (**a**) Urinary dopamine level in wild-type (WT) and *Mate1*-knockout (KO) mice during acute saline infusion. ** *p* < 0.01 compared to WT mice (*n* = 3 for each group); (**b**) Renal dopamine level in WT and *Mate1* KO mice after acute saline infusion. * *p* < 0.05, compared to WT mice (*n* = 3 for each group); (**c**) Urinary Na^+^ excretion level of WT (*n* = 3) and *Mate1* KO mice (*n* = 4) during acute saline infusion. * *p* < 0.05 compared to WT mice; ^†^ *p* < 0.05 and ^††^ *p* < 0.01 compared to control period; (**d**) Urinary K^+^ excretion level of WT (*n* = 3) and *Mate1* KO mice (*n* = 4) during acute saline infusion. ^†^ *p* < 0.05 compared to the control period; (**e**) Urinary Cl^−^ excretion level of WT (*n* = 3) and *Mate1* KO mice (*n* = 4) during acute saline infusion. * *p* < 0.05 compared to WT mice; ^†^ *p* < 0.05 and ^††^ *p* < 0.01 compared to the control period; (**f**) Urinary volume of WT (*n* = 3) and *Mate1* KO mice (*n* = 4) during acute saline infusion. ** *p* < 0.01 compared to WT mice; ^†^ *p* < 0.05 and ^††^*p* < 0.01 compared to the control period; (**g**) Ratio of total body water weight to total body weight of intact WT (*n* = 7) (Ο) and *Mate1* KO mice (●) (*n* = 8). Bars indicate the mean values. ** *p* < 0.01 compared to WT mice.

Next, to clarify the MATE transporter-mediated renal tubular secretion of dopamine and consequent promotion of renal Na^+^ excretion in vivo, we carried out acute saline volume expansion experiments in wild-type and *Mate1*-knockout mice. This is because intravenous saline infusion is known to accelerate dopamine synthesis in the kidney and promotes urinary Na^+^ excretion [28]. The results revealed that urinary dopamine was barely detectable in *Mate1*-knockout mice (Figure 2a). The renal dopamine level was 1.5-fold higher in the *Mate1*-knockout mice than it was in their wild-type counterparts after acute saline volume expansion (Figure 2b). These results show that the urinary dopamine excretion was impaired by *Mate1* knockout and explained why dopamine accumulates in the kidneys.

Because renally-synthesized dopamine is a natriuretic catecholamine, we examined the effect of urinary dopamine depletion in *Mate1*-knockout mice. Volume expansion induced a 12.3-fold increase in urinary Na^+^ excretion in wild-type mice, whereas that in the *Mate1*-knockout group decreased to a 1.5-fold increase (Figure 2c). The urinary K^+^ excretion slightly increased by 1.7-fold in the wild-type mice (Figure 2d) compared to the change in Na^+^ excretion (Figure 2c). The changes in urinary Cl^−^ excretion between the control and volume expansion groups were similar to that of urinary Na^+^ excretion. Specifically, there were 10.4- and 2.7-fold increases in urinary Na^+^ excretion in the wild-type and *Mate1*-knockout mice, respectively (Figure 2e). Furthermore, the urinary volume increased by 5.7-fold and 1.7-fold in the wild-type mice in *Mate1*-knockout mice, respectively (Figure 2f). Together, these results indicate that *Mate1* knockout impairs natriuresis because excretion of dopamine into the tubular lumen is impaired.

Considering that *Mate1* knockout impairs natriuresis, we assessed whether it also caused fluid retention. We discovered that the ratio of total body water weight to total body weight of the *Mate1*-knockout mice was significantly higher than that of wild-type mice (*p* < 0.01; Figure 2g). This result indicated that fluid retention occurred in *Mate1* knockout mice. The body weights were similar between wild-type and *Mate1*-knockout animals (Table 2). Furthermore, the blood Na^+^, K^+^ and glucose levels were weakly changed by *Mate1* knockout, but these results were statistically significant (Table 2).

**Figure 3:**
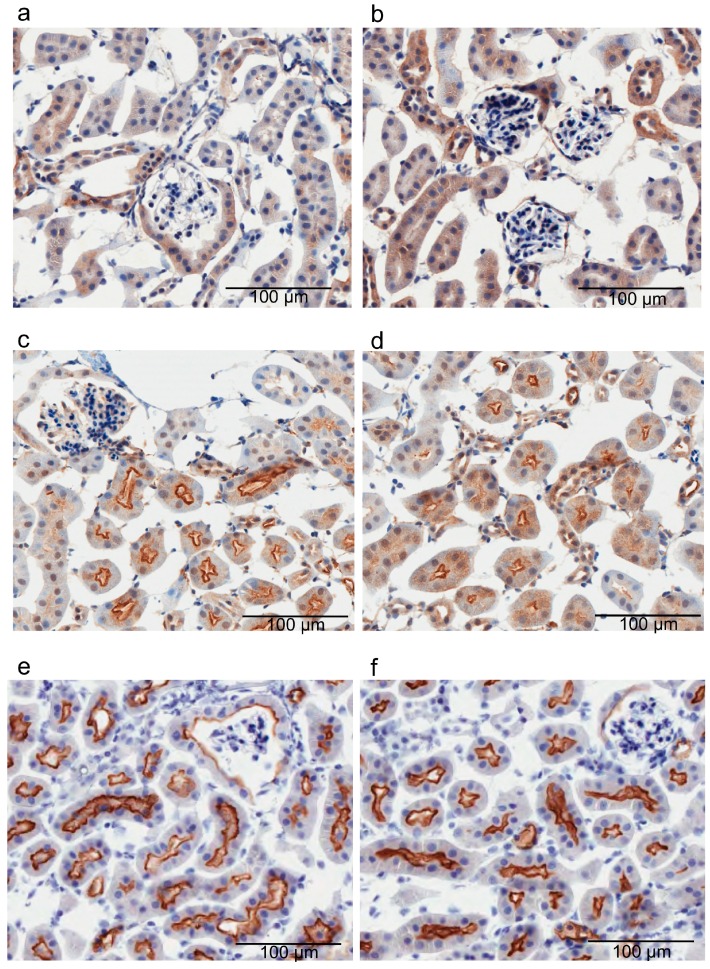
Expression of D1-like receptors and Na^+^/H^+^ exchanger (NHE)3 in wild-type (WT) and *Mate1-*knockout (KO) mouse kidneys. The scale bar represents 100 µm. Immunohistochemistry of renal D1 receptor (**a**,**b**), D5 receptor (**c**,**d**), NHE3 (**e**,**f**) in WT (**a**,**c**,**e**) and *Mate1*-KO (**b**,**d**,**f**) mice, respectively.

To examine whether *Mate1* knockout alters the dopamine receptor localization, we examined the expression of D1 and D5 (D1-like receptors) in mouse kidneys because D1-like receptors are responsible for over 50% of the dopamine-induced natriuresis [1,5]. Immunohistochemical analysis revealed that localization of both receptor subtypes was similar in the kidneys of the wild-type and *Mate1*-knockout mice (Figure 3a–d). We also examined the expression of the NHE3 transporter because it plays a dominant role in Na^+^ reabsorption [29], and we discovered that it was also similarly localized in the kidneys of wild-type and *Mate1*-knockout mice (Figure 3e,f).

### 2.3. Effects of Imatinib on Urinary Dopamine and Na^+^ Excretion in Mice

**Figure 4:**
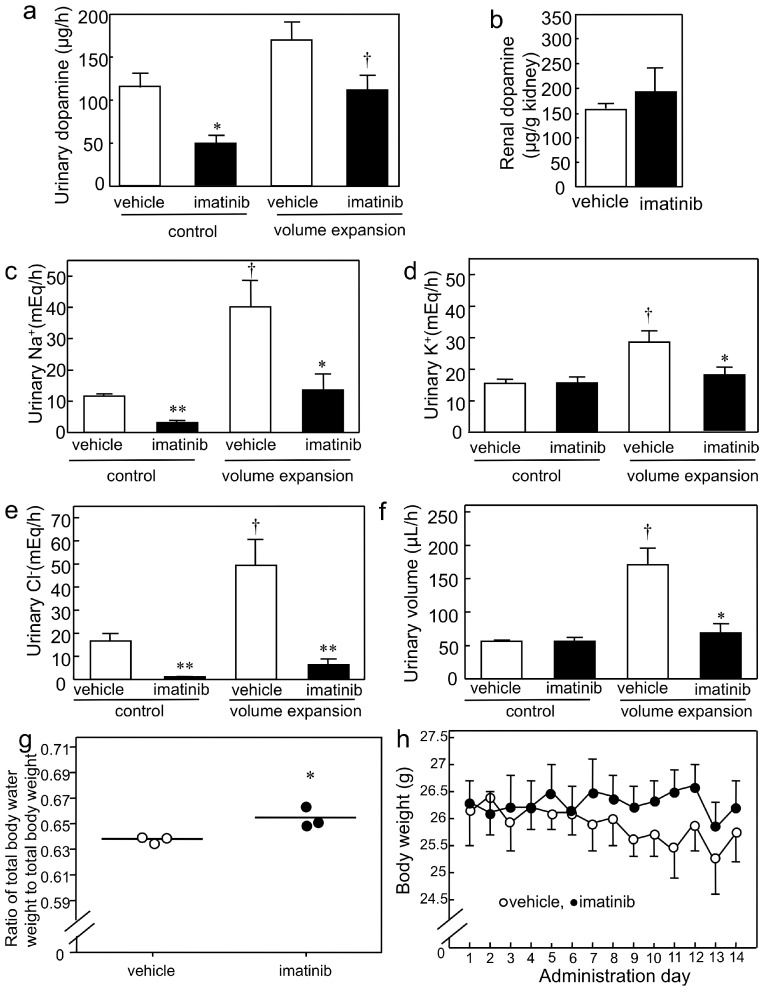
Effect of imatinib on natriuresis resulting from renally-synthesized dopamine and fluid retention in mice. (**a**) Urinary dopamine level of vehicle- and imatinib-treated WT mice during acute saline infusion. * *p* < 0.05 and ^†^ *p* < 0.05 compared to vehicle-treated WT mice and control period, respectively, *n* = 3 for each group; (**b**) Renal dopamine level of vehicle- and imatinib-treated WT mice after acute saline infusion, *n* = 3 for each group; (**c**) Urinary Na^+^ excretion level of vehicle- and imatinib-treated WT mice during acute saline infusion (*n* = 3 and 4 for vehicle and imatinib groups, respectively). * *p* < 0.05 and ** *p* < 0.01 compared to vehicle-treated WT mice, ^†^ *p* < 0.05 compared to control period; (**d**) Urinary K^+^ excretion levels of vehicle- and imatinib-treated WT mice during acute saline infusion (*n* = 3 and 4 for vehicle and imatinib groups, respectively). * *p* < 0.05 and ^†^ *p* < 0.05 compared to vehicle-treated WT mice and control period, respectively; (**e**) Urinary Cl^−^ excretion level of vehicle- and imatinib-treated WT mice during acute saline infusion (*n* = 3 and 4 for vehicle and imatinib groups, respectively). ** *p* < 0.01, compared to vehicle-treated WT mice; ^†^ *p* < 0.05, compared to control period; (**f**) Urinary volume of vehicle- and imatinib-treated WT mice during acute saline infusion (*n* = 3 and 4 for vehicle and imatinib groups, respectively). * *p* < 0.05 and ^†^ *p* < 0.05 compared to vehicle-treated WT mice and control period, respectively; (**g**) Ratio of total body water weight to total body weight of vehicle- and imatinib-treated wild-type (WT) mice (Ο and ●, respectively, *n* = 3 per group). Bars indicate the mean values. * *p* < 0.05, compared to vehicle-treated WT mice; (**h**) Body weight change of vehicle- and imatinib-treated WT mice (Ο and ●, respectively, *n* = 3 per group).

Since imatinib inhibits MATE [21] and edema is a common side effect in patients treated with imatinib [30], we tested whether imatinib inhibits urinary dopamine excretion in mice. We discovered that the urinary dopamine excretion was significantly decreased in the imatinib-treated group than it was in the vehicle-treated group during the control period (i.e., at a moderate saline infusion rate; Figure 4a). The renal dopamine levels of the imatinib-treated group after acute saline volume expansion were similar to those of the vehicle-treated groups (Figure 4b). During the control period, imatinib administration decreased the urinary Na^+^ and Cl^−^ excretion (Figure 4c,e); in addition to these ions, it also decreased K^+^ and urinary volume during the volume expansion (Figure 4c–f). In the vehicle-treated group, there was a significant increase in urinary Na^+^, K^+^ and Cl^−^ excretion (3.5-, 1.8- and 3.0-fold; Figure 4c–e, respectively) during the volume expansion treatment. Furthermore, imatinib administration increased the ratio of total body water weight to total body weight of mice (Figure 4g) without altering their body weights (Figure 4h). The blood total CO_2_ level was lower in the imatinib- than it was in vehicle-treated mice (Table 3). These results indicate that imatinib impaired urinary dopamine excretion and natriuresis.

### 2.4. Imatinib Inhibits MATE-Dependent Uptake of Dopamine

To determine whether imatinib affects MATE-mediated dopamine transport, we carried out [^3^H]dopamine uptake experiments in the presence of imatinib. The results revealed that dopamine transport activities of hMATE1, hMATE2-K and mMATE1 were inhibited by imatinib in a dose-dependent manner, and the calculated IC_50_ values were 1.1, 13.8 and 100.6 µM for hMATE1, hMATE2-K and mMATE1, respectively (Table 4). In the evaluation of ratio of total body water weight to total body weight, imatinib reached a concentration of 92.1 and 59.0 µM in the plasma and kidney, respectively. In the acute saline volume expansion experiments, plasma and kidney concentrations were 65.6 and 347.1 µM, respectively. We calculated the dopamine transport activity of mMATE1 relative to the renal imatinib concentration. There was a 37% reduction in mMATE1 activity at 59.0 µM imatinib and a 77% reduction at 347.1 µM. Taken together, these results support the hypothesis that imatinib inhibits natriuresis by disturbing the MATE-mediated dopamine secretion into the tubular lumen and consequently causes fluid retention.

We also examined the effects of the tyrosine kinase inhibitors, dasatinib and nilotinib, on MATE-dependent dopamine uptake. We found that dasatinib inhibited MATE-dependent [^3^H]dopamine uptake with IC_50_ values of 7.1 µM for hMATE1, 4.1 µM for hMATE2-K and 106.2 µM for mMATE1 (Table 4). However, IC_50_ values could not be determined for nilotinib because of its poor solubility (Table 4).

## 3. Discussion

A previous study designed to elucidate the mechanism of renal dopamine secretion used a porcine-derived renal epithelial cell line (LLC-PK_1_) [31]. This line possesses proximal tubule cell-like properties and releases dopamine synthesized from l-DOPA [31,32]. Because both cocaine (a non-selective competitive inhibitor of monoamine transporters) and GBR-12909 (a specific dopamine transporter inhibitor) failed to inhibit outward transfer of synthesized dopamine in LLC-PK_1_, it was postulated that monoamine transporters are not involved in the secretion of renally-synthesized dopamine into the tubular lumen [31]. The inside of the cells is generally negatively charged. Therefore, uptake of organic cations such as dopamine from the circulation into PTCs is driven by a downhill gradient through a potential-driven facilitated diffusion process. In contrast, the secretion of organic cations into the tubular lumen from PTCs goes against an uphill charge gradient [33]. Uphill transport requires a concomitant driving force, which is provided by an oppositely-directed H^+^ gradient in the case of MATE and by adenosine triphosphate (ATP) in the case of P-glycoprotein (P-gp). P-gp transports large and hydrophobic cationic drugs, including digoxin, anticancer agents, cyclosporine and tacrolimus [34]. An in vitro study showed that vesicular monoamine transporter (VMAT) 1 sequestered renally-synthesized dopamine, and exocytosis was involved in dopamine release from PTCs [35]. However, the effect of a VMAT inhibitor on dopamine release was mild [35]. A vesicle uptake study revealed that a H^+^/organic cation antiporter system is present in the apical membrane of LLC-PK_1_ cells [36]. Furthermore, urinary dopamine excretion is defective in *Mate1*-knockout mice (Figure 2a). Therefore, MATE, rather than P-gp, exocytosis or monoamine transporters, mediates dopamine secretion into the tubular lumen. 

In the human kidney, both hMATE1 and hMATE2-K appear important for dopamine secretion into the PTL, since the levels of their respective mRNAs were similar in this tissue (approximately 80 and 60 amol/µg total RNA, respectively) [13]. Furthermore, the [^3^H]dopamine transport activity of hMATE1 and hMATE2-K was similar (Table 1). Functionally null mutants with complete loss of hMATE1 and hMATE2-K transport activities have been identified [37]. Only heterozygous carriers have been identified, and the allelic frequency of the loss-of-function mutant is low, at 0.6% for hMATE1 and 1.7% for hMATE2-K [37]. Heterozygous MATE variants do not affect oral clearance of metformin [38]. Therefore, MATE transporters may play important physiological roles, and hMATE1 and hMATE2-K may be considered to compensate for each other.

In PTCs, dopamine receptors are expressed in the brush border membranes and basolateral membranes [39]. However, the degree of contribution of each receptor expressed in brush border membrane or basolateral membrane to the Na^+^ excretion effect remains unclear. The urinary Na^+^ level in the volume expansion period was 38.9 and 9.8 µ equivalent (Eq)/h for the wild-type and *Mate1*-knockout mice, respectively (Figure 2c), while the urinary dopamine was barely detectable in *Mate1*-knockout mice (Figure 2a). Therefore, it is likely that the 29.1 µEq/h (75%) difference in urinary Na^+^ between the wild-type and *Mate1*-knockout mice was due to differences in dopamine secretion into the tubular lumen via apical dopamine receptors.

The *K*m value of dopamine uptake by mMATE1 (0.53 ± 0.08 mM; Table 1) was very similar to the renal level in intact wild-type mice (approximately 100 ng/mg or 0.65 mM) [40]. Additionally, the renal dopamine level in wild-type mice after volume expansion treatment was approximately 150–200 µg/g in kidney or 0.98–1.3 mM (Figure 2b and Figure 4b). Therefore, mMATE1 transport velocity is not saturated at in vivo dopamine concentrations. Thus, volume expansion treatment increased mouse renal dopamine concentration, which subsequently led to the nonlinear region of the mMATE1 dopamine transport velocity.

In the human kidney PTCs, various organic cations (e.g., tetraethylammonium, metformin, oxaliplatin, varenicline, cimetidine and creatinine) are taken up from the circulation by the membrane potential-dependent OCT2, a basolateral transporter, and secreted into the tubular lumen by MATE1 and MATE2-K, which are both apical transporters [16,41,42]. Dopamine is known as a substrate of OCT2 [42], and in the present study, we showed that it is also a substrate of MATE. This suggests a model in which OCT2 mediates dopamine uptake from the circulation into PTC membrane in a membrane potential-dependent manner, whereas MATE1 and MATE2-K mediate tubular secretion of the renally-synthesized dopamine into the lumen in a direction opposite to the H^+^ gradient.

Hepatic metabolism is the major route of imatinib elimination, and the cytochrome P-450 (CYP) 3A isoenzyme subfamily breaks down imatinib via oxidative reactions [43]. Imatinib is also a substrate of P-gp and the breast cancer resistance protein (BCRP) [44]. Imatinib inhibits creatinine and metformin transport by the organic cation transporter (OCT) and MATE [21,45]. In the current study, we found that imatinib also inhibited MATE-mediated dopamine transport. Monitoring of the pharmacokinetics and pharmacodynamics of tyrosine kinase inhibitors is critical during the clinical administration of these drugs. The plasma trough concentration (*C*_trough_) of imatinib must be >1000 ng/mL (2 µM) to achieve clinical efficacy in patients with chronic myelocytic leukemia (CML) or gastrointestinal stromal tumors [46]. Because the clinical plasma levels of imatinib are similar to the IC_50_ values of hMATE1 and hMATE2-K (Table 4), it is reasonable to assume that imatinib will inhibit MATE-dependent dopamine secretion into the tubular lumen in patients. In the case of dasatinib, the *C*_trough_ should not exceed 2.5 ng/mL (0.005 µM) in order to meet safety requirements when used to treat the chronic phase in patients with CML [46]. The recommended *C*_trough_ of nilotinib in patients with CML is >761 ng/mL (1.4 µM) [46]. In contrast to imatinib, the recommended clinical plasma concentrations of dasatinib and nilotinib are much lower than the IC_50_ values of these drugs concerning hMATE1- and hMATE2-K-mediated dopamine uptake (Table 4). Thus, we can infer that patients taking imatinib experience edema more frequently than those taking dasatinib and nilotinib because of the differential IC_50_ values that these drugs have for MATE inhibition. Edema is the most common side effect associated with imatinib treatment (it is observed in 55.5% of patients), and the risk factors are a high dosage, or plasma concentration, or both [30,47]. Dietary restriction of salt improves edema symptoms in some patients [30]. Patients with edema usually continue to take imatinib without dose reduction because, in most cases, the accompanying edema is superficial and presents with a mild to modest severity. However, edema can contribute to poor patient adherence, which is a key factor for achieving a stable major molecular response [48,49]. Periorbital edema is the most frequent type of imatinib-induced water retention, and some patients require surgical intervention to recover visual field defects [47]. The etiology of imatinib-induced edema is still unknown, which thwarts the development of novel dosing or adjuvant strategies to ameliorate these effects.

**Figure 5:**
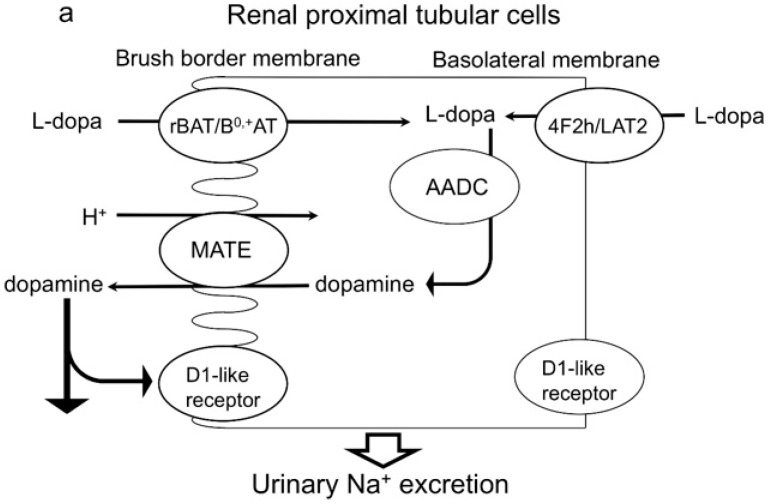

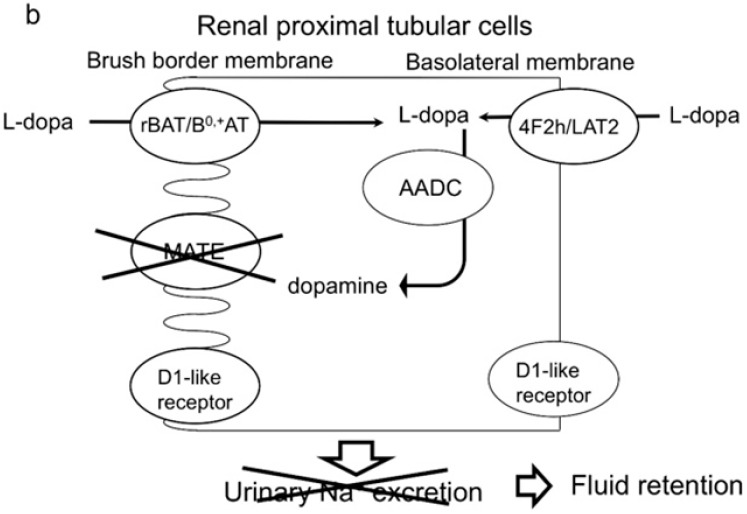
Mechanism of the secretion of dopamine synthesized in the kidney into proximal tubular cells. (**a**) MATE mediates dopamine secretion into the apical lumen and, consequently, promotes urinary Na^+^ excretion by acting on dopamine receptors that are expressed in multiple nephron sites; (**b**) MATE dysfunction inhibits dopamine secretion into the apical lumen and, consequently, triggers fluid retention by inhibiting urinary Na^+^ excretion. Related to B°^,+^ amino acid transporter/ B°^,+^-type amino acid transporter dimer, rBAT/B°^,+^AT; 4F2 heavy chain/l-type amino acid transporter 2 dimer, 4F2h/LAT2; multidrug and toxin extrusion, MATE; aromatic amino acid decarboxylase, AADC; l-dihydroxyphenylalanine, l-DOPA; D1 receptor and D5 receptor, D1-like receptor.

In conclusion, our results show that MATE mediated dopamine secretion and, consequently, promoted natriuresis (Figure 5a), and thus, its dysfunction is a risk for fluid retention (Figure 5b). Therefore, urinary dopamine has potential usefulness as a noninvasive index of the MATE transporter activity in the kidney.

## 4. Materials and Methods

### 4.1. Cell Culture

HEK293 cells (American Type Culture Collection, Manassas, VA, USA; ATCC CRL-1573) were cultured in complete medium consisting of Dulbecco’s Modified Eagle’s Medium (DMEM) (Wako Pure Chemical Industries, Osaka, Japan) with 10% fetal bovine serum (FBS, Life Technologies Corporation, Carlsbad, CA, USA) in an atmosphere of 5% CO_2_/95% air at 37 °C. For transient expression, HEK293 cells were seeded on 24-well (2 × 10^5^ cells/well) poly-d-lysine-coated plates (BD Biocoat, Franklin Lakes, NJ, USA) and transfected with hMATE1, hMATE2-K and mMATE1 cDNA containing plasmid vectors and empty vectors (pcDNA3.1(+) for hMATE1 and hMATE2-K, pFLAG for mMATE1) by using the LipofectAMINE 2000 Reagent (Life Technologies Corporation), according to the manufacturer’s instructions. The cells were used for uptake experiments at 48 h after transfection, while the empty vector-expressing cells were used as control.

### 4.2. Dopamine Uptake Experiments

The MATE-expressing HEK293 cells were preincubated with 0.2 mL of incubation medium (145 mM NaCl, 3 mM KCl, 1 mM CaCl_2_, 0.5 mM MgCl_2_, 5 mM d-glucose, 5 mM HEPES, 1 mM ascorbic acid and 10 µM U0521) containing 30 mM ammonium chloride (pH 7.4) for 20 min at 37 °C to induce intracellular acidification, because MATE moves solutes against a H^+^ gradient [11]. After the preincubation medium was removed, 0.2 mL of incubation medium containing 82 nM (1.13 µ curie (Ci)/mL) of [^3^H]dopamine (NET131250UC, PerkinElmer, Waltham, MA, USA) were added. In the *cis*-inhibition experiments, various concentrations of imatinib, dasatinib and nilotinib were added in addition to [^3^H]dopamine. Following the indicated incubation times at 37 °C, the medium containing [^3^H]dopamine was aspirated, and the monolayers were rapidly rinsed twice with 1 mL of ice-cold incubation medium without 10 µM U0521. The cells were dissolved in 0.5 mL of 0.5 normality (N) sodium hydroxide (NaOH), and then, the radioactivity in 200 µL was determined using liquid scintillation counting following addition to 2 mL of Ultima Gold (PerkinElmer). The protein content of each NaOH solution was determined using a Bio-Rad Protein Assay Kit (Bio-Rad Laboratories, Hercules, CA, USA) by using bovine γ-globulin as a standard. The *K*m and *V*_max_ values were calculated from the saturation curve using the Michaelis–Menten equation, *V* = *V*_max_[*S*]/(*K*m + [*S*]), with Kaleidagraph Version 4.00 (Synergy Software, Reading, PA, USA) after the nonspecific uptake values were subtracted. The nonspecific uptake values were calculated from the linear model obtained from 1 and 5 mM [^3^H]dopamine uptake values in the presence of 5 mM tetraethylammonium. The IC_50_ values were calculated from the inhibition plots using the equation, *V* = *V*_0_/[1 + ([I]/IC_50_)^n^], with Kaleidagraph Version 4.00, where *V* and *V*_0_ are the uptake amounts of [^3^H]dopamine in the presence and absence of the inhibitor, respectively, [*I*] is the concentration of the inhibitor and n is the Hill coefficient.

### 4.3. Animals

Mice aged 17 weeks or younger were used in all experiments. The male wild-type mice and Mate1-knockout mice were on C57BL/6NCrSlc and C57BL/6 genetic backgrounds, respectively [14]. All of the mice were treated according to the Fundamental Guidelines for Proper Conduct of Animal Experiment and Related Activities in Academic Research Institutions under the jurisdiction of the Ministry of Education, Culture, Sports, Science and Technology in Japan. MedKyo12133, 26 March 2012, Kyoto University Animal Care and User Committee. 070661, 16 May 2008, Kyoto University Safety Committee for Recombinant DNA Experiments.

### 4.4. Immunohistochemical Analysis

The mice were anesthetized, and their kidneys were perfused via the left ventricle, first with saline containing 25 U/mL of heparin, followed by 4% paraformaldehyde in phosphate-buffered saline (PBS). The fixed tissues were embedded in OCT compound (Sakura Finetechnical, Tokyo, Japan), frozen rapidly in liquid nitrogen; 6 µm-thick sections were cut and then incubated with 10 mM of citrate buffer pH 6 (D1 and D5) or 1 mM of EDTA-2Na buffer pH 9 (NHE3) at 95 °C for 40 min for antigen retrieval. After washing with PBS, the sections were incubated with 3% skim milk in PBS at room temperature for 15 min. Following another PBS wash, the sections were incubated at 4 °C overnight with specific antiserum specific for D1 (ab20066; Abcam, Cambridge, MA, USA) (1:4500 dilution), D5 (GTX77969; GeneTex, Irvine, CA, USA) (1:1600 dilution) or anti-NHE3 antibody (NHE31-A; ALPHA DIAGNOSTIC, San Antonio, TX, USA) (1:1000 dilution). Following three PBS washes (5 min each), the sections were incubated with hydrogen peroxide (0.3% in methanol) at room temperature for 30 min. After three additional PBS washes (5 min each), the sections were incubated with EnVision Horseradish Peroxidase (K4003; DAKO, Hamburg, Germany) at room temperature for 30 min. After washing with distilled water, the nuclei were stained with hematoxylin, and the images were captured using an Aperio (Leica, Wetzlar, Germany).

### 4.5. Determination of Fluid Content

The imatinib- (200 mg/kg oral administration, for 14 days) and vehicle-treated (lactated Ringer’s solution) wild-type, intact wild-type and intact [17] *Mate1*-knockout mice were anesthetized with isoflurane. Blood was collected from the inferior vena cava, and the chemical parameters were measured using a CHEM8+ cartridge and the i-STAT analyzer (Abbott, Abbott Park, IL, USA). Then, the mouse carcasses were heated in an oven at 60 °C over 14 days or until there was no further change in weight loss on 2 consecutive days. The difference between the live mouse weight and dried carcass weight was used to determine the fluid content of each mouse [50]. The total body water weight to total body weight ratio was calculated as follows: (living mouse weight-dried mouse weight)/living mouse weight. The mouse body weight was measured using an ANDGX-2000 (A&D Company, Tokyo, Japan), and the body weight was measured just before oral administration of imatinib or the vehicle.

### 4.6. Acute Saline Volume Expansion Experiments

For the saline infusion, a catheter was inserted into the femoral vein with polyethylene tubing (Intramedic PE-10; BD Biosciences, San Jose, CA, USA) in mice anesthetized with 50 mg/kg of pentobarbital. To collect the urine, the urinary bladder was catheterized with SP-31 tubing (Natsume Seisakusho, Tokyo, Japan). The saline was infused using an automatic infusion pump (Harvard Apparatus, Inc., Holliston, MA, USA) at 0.24 mL/h for 30 min in the stabilization period, 0.12 mL/h for 60 min in the control period and 3 mL/h for 30 min in the volume expansion period. Following the latter period, the infusion pump was stopped, and the mice were then left for 30 min before the kidney samples were collected. The urinary electrolyte levels of the mice were measured using an ion-selective electrode (cobas6000, Roche, Basel, Switzerland). For dopamine detection, 1 N HCl was added to the urinary collection tube, and 0.2 N HClO_4_ was added to kidney collection tube to prevent dopamine degradation. In the imatinib inhibition study, 500 mg/kg imatinib dissolved in lactated Ringer’s solution were administered orally to wild-type mice 3 h or immediately before saline infusion.

### 4.7. Sample Preparation

The catecholamines in the urine reagent kit (CHROMSYSTEMS, Munich, Germany) were used for dopamine sample preparation according to the manufacturer’s instructions with some modifications. For urinary dopamine detection, the urine was first diluted to a total volume of 300 µL with 1% 6 N HCl, and then, 50 µL of the internal standard (DHBA) were then added, followed by 6 mL of neutralization buffer. We applied 600 µL elution buffer to the column in a final step and added formic acid to the collected eluate. Forty microliters of sample was injected into the LC-MS/MS system after being filtrated with a filter (06543-04, Nacalai Tesque, Kyoto, Japan). The kidney homogenate was diluted 5- or 6-fold with 1% 6 N HCl. After centrifugation, 300 µL of the supernatant were subjected to the same procedure used for the urine sample above.

For imatinib detection, the mouse plasma and kidney homogenate samples were diluted 40× and 400× with saline, respectively, and 10 µL of roscovitine solution (1 µg/mL) were added to 100 µL of the diluted samples. Then, the samples were deproteinized with 200 µL of acetonitrile for 10 min with agitation. After centrifugation (14,680 rpm, 15 min) using the Centrifuge 5424 (Eppendorf, Hamburg, Germany), the supernatant was diluted with 200 µL of 0.2% formic acid, and 1 µL of the sample was injected into the LC-MS/MS system after filtration using a filter (06541-24, Nacalai Tesque, Kyoto, Japan).

### 4.8. Liquid Chromatography-Tandem Mass Spectrometry (LC-MS/MS)

The dopamine and imatinib were analyzed by using liquid chromatography-tandem mass spectrometry (LC-MS/MS). A liquid chromatography system consisting of a Prominence series chromatograph (Shimadzu, Kyoto, Japan) coupled to an API4000 triple-quadrupole tandem mass spectrometer (AB SCIEX, Foster City, CA, USA) was used for dopamine detection, and an Eksigent ultra-performance liquid chromatography (AB SCIEX) coupled to a QTRAP4500 triple-quadrupole tandem mass spectrometer (AB SCIEX) was used for imatinib detection. An Inertsil ODS-3 (GL Sciences, Tokyo, Japan) was used for chromatographic separation. The mobile phase was composed of 0.1% formic acid and acetonitrile containing 0.1% formic acid. For dopamine detection, gradient elution was carried out at a flow rate of 0.25 mL/min. 3,4-dihydroxybenzylamine (DHBA) was used as an internal standard. The detection was carried out in the multiple reaction monitoring mode by monitoring ion transitions of *m*/*z* 154.1 → 91.2 for dopamine and *m*/*z* 140.1 → 77.1 for DHBA. For imatinib detection, gradient elution was carried out at a flow rate of 0.2 mL/min. Roscovitine was used as an internal standard. Detection was carried out in the multiple reaction-monitoring mode by monitoring ion transitions of *m*/*z* 494.171 → 393.900 for imatinib and *m*/*z* 355.047 → 233.100 for roscovitine.

### 4.9. Statistical Analysis

All data are expressed as the mean ± standard error (S.E.) and were analyzed statistically using the unpaired *t*-test.

## Figures and Tables

**Table 1:** Kinetic parameters of [^3^H]dopamine uptake in HEK293 cells transiently expressing human multidrug and toxin extrusion (hMATE) 1, hMATE2-K and mouse (m) MATE1.

Kinetic Parameters	hMATE1	hMATE2-K	mMATE1
*K*m (mM)	0.56 ± 0.18 *	2.48 ± 0.65 ^‡^	0.53 ± 0.08
*V*_max_ (nmol·mg·protein^−1^·min^−1^)	3.71 ± 0.15 *	7.69 ± 1.12	8.73 ± 0.08 ^††^
*V*_max_/*K*m (µL·mg·protein^−1^·min^−1^)	7.70 ± 1.67	3.44 ± 0.78 ^‡‡^	17.20 ± 2.72 ^†^

Data represent the mean ± standard error (S.E.) of three separate experiments. * *p* < 0.05, hMATE1 vs. hMATE2-K unpaired *t*-test. ^‡^ *p* < 0.05, ^‡‡^ *p* < 0.01, hMATE2-K vs. mMATE1 unpaired *t-*test. ^†^ *p* < 0.05, ^††^ *p* < 0.01, hMATE1 vs. mMATE1 unpaired *t-*test.

**Table 2:** Blood parameters and body weight of wild-type and *Mate1-*knockout mice.

Blood Parameters and Body Weight	Wild-Type Mice	*Mate1*-Knockout Mice
Na^+^ (mmol/L)	147.0 ± 0.2	149.1 ± 0.6 **
K^+^ (mmol/L)	4.0 ± 0.1	3.8 ± 0.0 *
Cl^−^ (mmol/L)	113.6 ± 0.4	114.3 ± 0.7
iCa (mmol/L)	1.2 ± 0.0	1.2 ± 0.0
tCO_2_ (mmol/L)	19.3 ± 0.5	19.6 ± 0.4
Glucose (mg/dL)	249.9 ± 10.0	182.4 ± 17.3 **
BUN (mg/dL)	25.1 ± 1.1	26.0 ± 2.6
Hct (%)	38.9 ± 0.5	37.6 ± 2.1
Hb (g/dL)(via Hct)	13.2 ± 0.2	12.8 ± 0.7
AnGap (mmol/L)	19.1 ± 0.6	19.9 ± 0.8
Body weight (g)	28.7 ± 0.4	29.1 ± 0.4

iCa, ionized calcium; tCO_2_, total carbon dioxide; BUN, blood urea nitrogen; Hct, hematocrit; Hb, hemoglobin; AnGap, anion gap. Values are the mean ± standard error (S.E.) for seven and eight wild-type and *Mate1-*knockout mice, respectively. * p < 0.05 and ** p < 0.01, significantly different from wild-type mice (unpaired *t*-test).

**Table 3:** Blood parameters and body weight of vehicle- and imatinib-treated mice.

Blood Parameters and Body Weight	Vehicle-Treated Mice	Imatinib-Treated Mice
Na^+^ (mmol/L)	147 ± 1.5	145.7 ± 1.5
K^+^ (mmol/L)	4.9 ± 0.3	4.5 ± 0.5
Cl^−^ (mmol/L)	115.0 ± 0.6	115.3 ± 1.2
iCa (mmol/L)	1.2 ± 0.0	1.3 ± 0.0
tCO_2_ (mmol/L)	25.0 ± 0.6	19.7 ± 1.5 *
Glucose (mg/dL)	284.0 ± 62.6	229.0 ± 39.3
BUN (mg/dL)	26.3 ± 0.3	21.0 ± 2.1
Hct (%)	39.7 ± 0.3	38.3 ± 2.0
Hb (g/dL)(via Hct)	13.5 ± 0.1	13.0 ± 0.7
AnGap (mmol/L)	13.0 ± 0.6	16.0 ± 1.5
Body weight (g)	26.6 ± 0.3	26.7 ± 0.8

iCa, ionized calcium; tCO_2_, total carbon dioxide; BUN, blood urea nitrogen; Hct, hematocrit; Hb, hemoglobin; AnGap, anion gap. Each value represents the means ± S.E. for three (vehicle- and imatinib-treated) mice. * p < 0.05, significantly different from vehicle-treated mice (unpaired *t*-test).

**Table 4:** Half-maximal inhibitory concentration (IC_50_) values of tyrosine kinase inhibitors on [^3^H]dopamine uptake mediated by hMATE1, hMATE2-K and mMATE1.

Transporter	Imatinib (µM)	Dasatinib (µM)	Nilotinib (µM)
hMATE1	1.1 ± 0.1	7.1 ± 0.6	>100
hMATE2-K	13.8 ± 4.4	4.1 ± 1.0	>100
mMATE1	100.6 ± 13.9	106.2 ± 7.0	>100

Imatinib and dasatinib data represent the mean ± S.E. of three separate experiments. Imatinib concentrations were 0, 0.3, 0.6, 1, 3 and 10 µM for hMATE1; 0, 2, 3, 6, 10 and 250 µM for hMATE2-K; and 0, 30, 60, 100, 150 and 250 µM for mMATE1. Dasatinib concentrations were 0, 0.3, 1, 3, 10 and 30 µM for hMATE1 and hMATE2-K and 0, 30, 60, 100, 150 and 200 µM for mMATE1.
